# *Nocardia transvalensis* Disseminated Infection in an Immunocompromised Patient with Idiopathic Thrombocytopenic Purpura

**DOI:** 10.1155/2016/3818969

**Published:** 2016-05-24

**Authors:** Jorge García-Méndez, Erika M. Carrillo-Casas, Andrea Rangel-Cordero, Margarita Leyva-Leyva, Juan Xicohtencatl-Cortes, Roberto Arenas, Rigoberto Hernández-Castro

**Affiliations:** ^1^Departamento de Posgrado y Educación Médica Continua, Instituto Nacional de Cancerología, Mexico; ^2^Departamento de Microbiología, Facultad de Medicina, UNAM, 04510 Coyoacán, MEX, Mexico; ^3^Departamento de Biología Molecular e Histocompatibilidad, Dirección de Investigación, Hospital General “Dr. Manuel Gea González”, 14080 Tlalpan, MEX, Mexico; ^4^Laboratorio de Microbiología Clínica, Instituto Nacional de Ciencias Médicas y Nutrición “Salvador Zubirán”, 14080 Tlalpan, MEX, Mexico; ^5^Departamento de Infectología, Hospital Infantil de México “Federico Gómez”, Dr. Márquez 162, Cuauhtémoc, 06720 Ciudad de México, DF, Mexico; ^6^Servicio de Micología, Hospital General “Dr. Manuel Gea González”, 14080 Tlalpan, MEX, Mexico; ^7^Departamento de Ecología de Agentes Patógenos, Hospital General “Dr. Manuel Gea González”, 14080 Tlalpan, MEX, Mexico

## Abstract

*Nocardia transvalensis* complex includes a wide range of microorganisms with specific antimicrobial resistance patterns*. N. transvalensis* is an unusual* Nocardia* species. However, it must be differentiated due to its natural resistance to aminoglycosides while other* Nocardia* species are susceptible. The present report describes a* Nocardia* species involved in an uncommon clinical case of a patient with idiopathic thrombocytopenic purpura and pulmonary nocardiosis. Microbiological and molecular techniques based on the sequencing of the 16S rRNA gene allowed diagnosis of* Nocardia transvalensis* sensu stricto. The successful treatment was based on trimethoprim-sulfamethoxazole and other drugs. We conclude that molecular identification of* Nocardia* species is a valuable technique to guide good treatment and prognosis and recommend its use for daily bases diagnosis.

## 1. Introduction

*Nocardia* species are Gram-positive ubiquitous, aerobic actinomycetes, saprophytic of soil, water, and organic matter.* Nocardia* genus is an opportunistic pathogen which may cause disease in immunocompromised or immunocompetent patients [1, 2]. The primary source of infection is through inhalation. However, more than 90% patients have underlying conditions compromising their cellular or humoral immunity.* N. transvalensis* must be differentiated due to its natural resistance to aminoglycosides, while other* Nocardia* species are typically susceptible such as* N. blacklockiae* and* N. wallacei* [3, 4].

*N*.* transvalensis* was first described by Pijper and Pullinger in 1927 as the causative agent of mycetoma of the foot in a South African patient [5]; since then, it has turned to be a cause of life threatening infections and other nonthreatening infections [4].* N. asteroides* and* N. brasiliensis* are common species recognized in clinical cases, while* N. transvalensis* sensu stricto is one of the least frequent* Nocardia* species. It has restricted susceptibility to cotrimoxazole, third-generation cephalosporin, imipenem, and linezolid.* N. transvalensis* is a pathogen rarely reported, particularly in a case of cystic fibrosis with chronic pulmonary infections [5], brain abscess [3], keratitis [4], ocular infections, HIV patients [6], mycetoma [1, 7], and pulmonary infections [8].

Here we present an uncommon report of pulmonary nocardiosis with haematological and neurological involvement caused by* N. transvalensis* in a patient with idiopathic thrombocytopenic purpura.

## 2. Case Description

**Figure 1:**
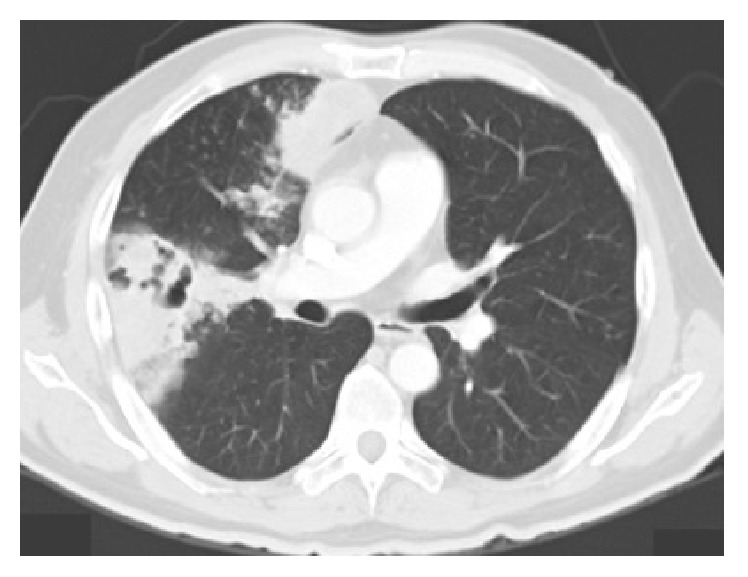
Axial computed tomography showing middle right lobe involvement.

**Figure 2:**
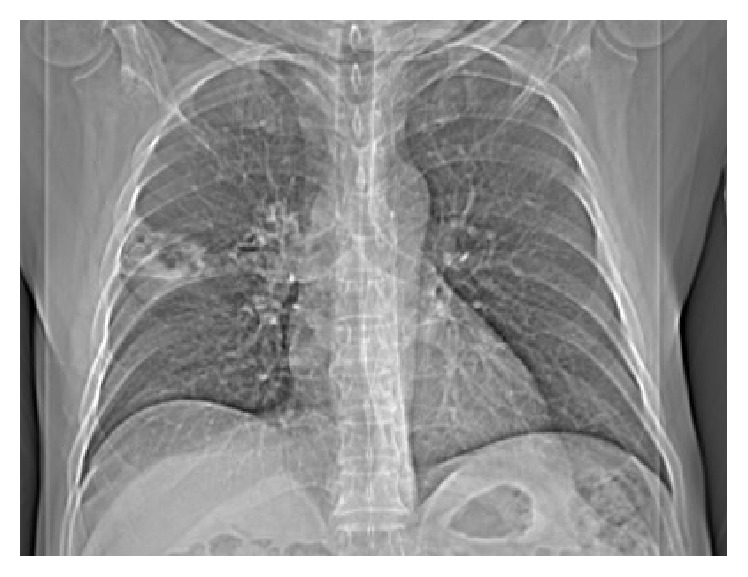
Chest computed tomographic scan at ER admission.

A 59-year-old male from Guerrero, Mexico, was referred to the National Cancer Institute at Mexico City, diagnosed with prostate adenocarcinoma (Gleason 8), and treated with flutamide (Androgen Receptor Inhibitor) plus goserelin (gonadotropin releasing hormone superagonist (GnRH agonist)). After exhaustive physical examination and thrombocytopenia (51/mm^3^), presumptive drug-induced idiopathic thrombocytopenic purpura was recognized. This condition was managed with prednisone 100 mg/day plus 6-mercaptopurine 50 mg/day, for 7 weeks. After 12 weeks at his hometown, the patient developed progressive respiratory insufficiency, diagnosed with right pneumonia and treated with oral levofloxacin (500 mg/daily). He was readmitted to the Cancer Institute with hyperglycaemia because of steroid use, and no bacterial isolation was obtained from the first sputum. One week later, the patient was received at the Emergency Room complaining of fever (39°C), dyspnea, cachexia, and haemoptysis. Clinically with right basal hypoventilation, the imaging studies (chest X-ray, followed by a thoracic computed tomography scan) revealed cavitated right middle lobe pneumonia, as well as lesions in other lung segments (Figures 1 and 2). The antimicrobial regimen was changed to ceftriaxone and clindamycin. In spite of mild improvement, the patient was submitted to a CT-guided lung biopsy. Gram and Ziehl-Neelsen stains were negative and until the fourth day waxy colonies were detected in blood agar and Sabouraud plates under incubation at 37°C and 5% CO_2_ atmosphere. Microscopically, weak Gram-positive, branched filamentous bacilli were observed. Trimethoprim-sulfamethoxazole (15 mg/kg/day) was the successful treatment. Almost immediately to diagnosis, the patient referred to right arm weakness, which reversed on the third day of treatment with trimethoprim-sulfamethoxazole. A Magnetic Resonance of the brain reported minimum changes in the meningeal layers, and the lumbar puncture showed moderate pleocytosis without recovering any microorganisms. A follow-up thoracic CT scan showed the resolution of more than half of lung involvement and trimethoprim-sulfamethoxazole was continued at home. The resolution of the idiopathic thrombocytopenic purpura was followed by tapering prednisone and 6-mercaptopurine (25 mg/48 h) for the next 6 and 12 months until discontinuation. One year after the episode and maintenance treatment with amoxicillin-clavulanate (40 mg/10 mg/kg/day) plus trimethoprim-sulfamethoxazole (15 mg/kg/day) for the* Nocardia* infection, the imaging studies confirmed the complete resolution of the lung pathology. Since then, the patient has not relapsed and the cancer treatment continued.

## 3. Materials and Methods

The genus identification was based on the Gram-positive stain of branching and filamentous bacilli, positive modified acid-fast stain, colonial morphology, and conventional biochemical reactions. Further species identification was based on sequencing of the 16S rRNA gene, using the primers Noc1 (5′-GCTTAACACATGCAAGTCG-3′) and Noc2 (5′-GAATTCCAGTCTCCCCTG-3′) [9]. The PCR product was purified with the QIAquick purification kit (Qiagen, Ventura, CA, USA), according to the manufacturer, and DNA sequences were determined with Taq FS Dye Terminator Cycle Sequencing Fluorescence-Based Sequencing and analysed on an Applied Biosystems 3730 DNA sequencing system (Foster City, CA, USA). The sequence of the 16S showed 100% homology with* N. transvalensis* accession number FJ516749.

## 4. Discussion

A marked increased number of human* Nocardia* infections have been reported worldwide since the decade of 1960. However,* N. transvalensis* is an infrequent pathogen and probably misdiagnosed. Pulmonary nocardiosis is a mayor clinical manifestation of* Nocardia* species infection and may pose a challenge when distinguishing it from tuberculosis. To the best of our knowledge, only few cases had been related involving* N. transvalensis* as the primary cause of acute or chronic infections. Predisposing factors of human nocardiosis are chronic obstructive pulmonary disease, bronchiectasis, pulmonary fibrosis, emphysema, asthma, neoplastic disease (thoracic or induced by potent immunosuppressant drugs), organ transplant (bone marrow or solid transplantation), or immunosuppressive therapy used in autoimmune diseases, cancer, diabetes mellitus, previous or concurrent tuberculosis, and advanced HIV infection (acquired immunodeficiency syndrome) [8].

The geographical differences among continents should be taken into account.* N. transvalensis* sensu stricto is common in Africa and absent in Asia.* N. wallacei* is the most frequent species isolated in the United States, whereas scarce information is available in the rest of America, maybe due to misdiagnosis or underestimation of human cases and difficulties in clinical and laboratory diagnosis.

The routine diagnosis has been microbiological culture and morphological features. However, the phenotypic identification has 37% misidentification of* Nocardia* species based on the 16S rRNA gene which discern among the* N. transvalensis* cluster and other species by two base insertions. Few reports have used molecular techniques to identify the involvement of* N. transvalensis* in clinical cases [4, 10]; nonetheless, its molecular identification is of great value as guidance for treatment choice [4]. The delay in the* Nocardia* species identification has clinical implications because these bacteria have been classified according to their antibiotic resistance patterns but share similar clinical manifestations; therefore, the treatment election may be hindered. In the current case, the use of trimethoprim-sulfamethoxazole (an antimicrobial of choice for nocardiosis) was the right choice in combination with other antimicrobials, which may include imipenem, amikacin, ceftriaxone, and amoxicillin-clavulanate.

## 5. Conclusion

The present report describes an uncommon* N. transvalensis* infection in an immunocompromised patient with idiopathic thrombocytopenic purpura. This work underlines the value of early diagnosis by microbiological culture combined with accurate molecular identification of the* Nocardia* species; in addition, sulfonamide extended use and the reduction in immunosuppression are key factors for good prognosis. We encourage the use of molecular identification at the species level for further understanding of the epidemiology, taxonomy, antimicrobial susceptibility, and clinical and epidemiological aspects of nocardiosis.
